# Developing Interprofessional Immigrant Health Education for Emergency Physicians

**DOI:** 10.5811/westjem.33576

**Published:** 2025-07-12

**Authors:** Leonardo Garcia Heglund, Katrin Jaradeh, Carolina Ornelas-Dorian, Nicholas Stark, Theresa Cheng, Christopher R. Peabody

**Affiliations:** * University of California, San Francisco, Department of Emergency Medicine, San Francisco, California; † Dignity Health - Mercy Medical Center, Department of Emergency Medicine, Merced, California

## Abstract

**Background:**

As of 2021, there were 47 million immigrants in the United States. Immigrant populations are uninsured at higher rates than US citizens, leading many to rely on emergency departments (ED) for their healthcare needs. However, emergency physicians (EP) often lack training on the unique challenges faced by this population, necessitating educational interventions.

**Methods:**

We implemented educational interventions for an urban emergency medicine residency program using Kern’s six-step approach for curriculum development to inform EPs of existing immigration-specific patient resources; teach social-medical-legal best practices with regard to asking, documenting, and sharing immigration-specific health information; and increase awareness of ED-relevant local policies. We developed three educational interventions.in collaboration with legal organizations, and community experts. To evaluate the success of these interventions we administered a pre- and post-survey to 64 EPs (36% of 178 targeted learners)

**Results:**

We found a significant increase in confidence and knowledge, with an average 5-point Likert scale score improvement of 1.47 (*P* < .001) in all responses and 1.40 *(P* < .001) in paired responses, and an improvement in test scores on the three knowledge-based questions of 30.66% (*P* < .001) in all responses and 33% (*P* = .02) in paired responses.

**Conclusion:**

This study highlights a model for interprofessional collaboration in curriculum development and the importance of a multipronged educational approach to improve the care of immigrants in the ED. The curriculum offers a framework for other EDs aiming to address healthcare inequities for this population. Future research can explore long-term knowledge retention, detailed educational tool utilization, and the impact on patients.

## BACKGROUND

As of 2023, the United States had about 47.8 million immigrants, with over half of them being non-citizens.^1^ Many studies have shown that immigrants are uninsured at disproportionately higher rates.^2^–^4^ As a result, many lack access to primary care.^4^,^5^ Lack of regular care renders this population more dependent on emergency departments (ED), and they are more likely to us EDs for less urgent healthcare needs.^6^ Emergency physicians (EPs are not consistently trained on the unique stressors faced by this patient community. For example, EPs have expressed a lack of education on immigration policies and unfamiliarity with how to interact with immigration officials.^7^,^8^ Having identified this gap (*Problem Identification*), there is a potential for immigrant health-focused educational interventions to address this problem. Using Kern’s six-steps for curriculum development of *Problem Identification, Targeted Needs Assessment, Objectives, Educational Strategies, Implementation*, and *Evaluation*, we implemented educational interventions to improve EPs’ knowledge of immigration-related resources, medical-legal practices, and health-systems policies.^9^

## OBJECTIVES

Following Kern’s six steps, we developed five measurable objectives. The EPs should be able to do the following: 1) identify existing immigration resources and their referral process; 2) execute hospital guidelines for responding to immigration law enforcement in the ED; 3) describe their responsibilities when caring for immigrants; 4) apply social-medical-legal best practices around asking, documenting, and sharing immigration-specific health information; and 5) define local immigration-related policies and their impact on the practice of emergency medicine (EM).

## CURRICULAR DESIGN

### Setting

We targeted an EM residency program associated with a Level I safety-net trauma center, pediatric hospital, and academic quaternary-care center. As identified in the *Problem Identification* step, the program lacked an immigrant health curriculum. The *Targeted Needs Assessment* included identifying the program’s 64 residents and 24 full-time residency faculty members as the targeted learners. The educational interventions were uploaded to E*Drive, the institution’s centralized, open-access clinical information hub (accessible at https://edrive.ucsf.edu/).^10^,^11^ The didactic was offered during the 2022–2023 and 2023–2024 academic years. The study was approved by the institutional review board (21-33252).

### Interprofessional Curricular Design

Our design process was iterative, involved community experts, and incorporated interprofessional expertise. Using *Targeted Needs Assessment* strategies of formal interviews and focus group discussions, we invited 35 local legal organizations to provide input on educating EPs on immigration resources. Ultimately, through snowball sampling, we conducted nine 30-minute, semi-structured interviews consisting of three legal organization leaders, two city public health employees, two social workers, and two non-EPs with expertise in working with immigrant populations, and two focus groups with two multi-practice legal working groups. From this targeted assessment we identified themes that defined the curricular *Objectives*.

**Figure 1:**
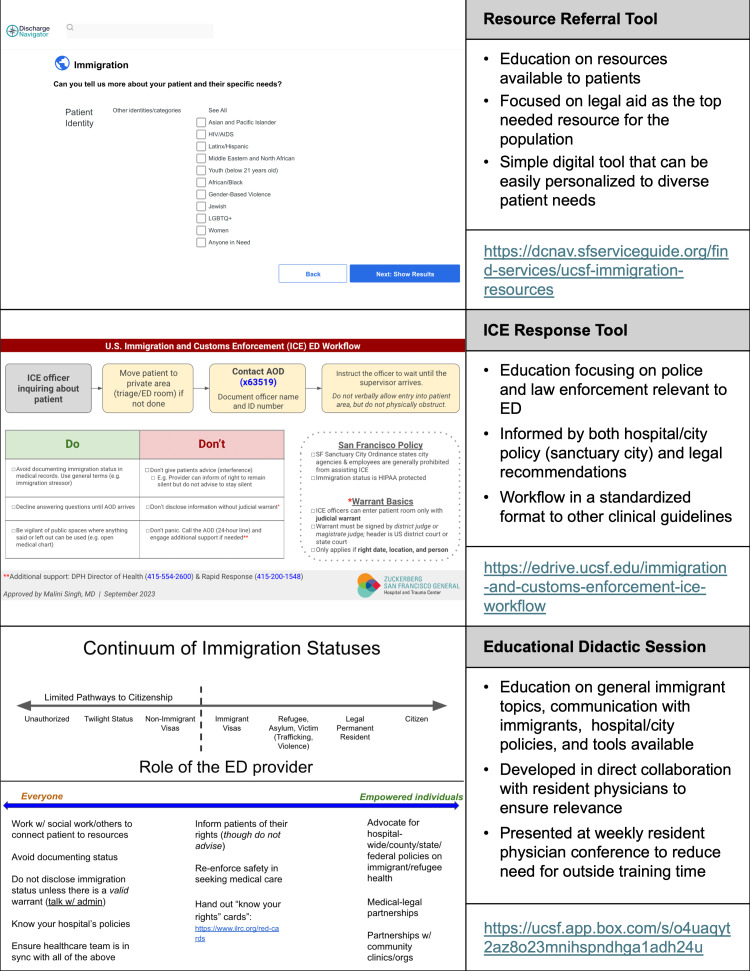
Educational strategies. *AOD*, administrator on duty; *ED*, emergency department; *ICE*, US Customs and Immigration Enforcement.

These partners also provided iterative feedback on the *Educational Strategies* (both “content and methods”) that were later designed. Prior to *Implementation*, local EP-leaders and administrators reviewed the curriculum. The *Educational Strategies* were guided by Kern’s principles of aligning with the objectives, involving multiple methods, and being resource-feasible. The interventions consisted of a customizable digital educational tool, an educational guideline handout, and an interactive didactic session. The strategies and their *Implementation* are detailed below (Figure 1).

### Discharge Community Resource Educational Tool

To address Objective 1, we created a digital educational tool for immigration-specific resources: the Immigration Discharge Navigator (IDN) (accessible at https://dcnav.sfserviceguide.org/find-services/ucsf-immigration-resources). The IDN consisted of a comprehensive list of immigration legal resources available to patients and ranked them based on demographic information and geographic proximity. By inputting these components into the IDN, EPs were able to view which organization best served their patient, with a description of the organization specialization (ie, providing wraparound care for HIV-positive immigrants), location, and patient-friendly discharge handouts in seven languages—English, Spanish, Tagalog, traditional Chinese, Vietnamese, Russian, and Arabic. Previous resources were decentralized, less accessible (often relying on social workers who are not always available), and were static lists, unable to be tailored based on patient-specific factors, such as subgroups (eg, pediatric, pregnant, and elderly populations).

Prior to *Implementation*, 15 regional immigrant-serving organizations reviewed the IDN, approving the *Educational Strategy* and the relevancy and accuracy of resources. They emphasized its ease of usability and reiterated the need for further interventions, such as training on discussing and documenting sensitive information in the ED.

### Immigration and Customs Enforcement Response Educational Guideline

To address Objective 2, we created an educational guideline detailing the EP’s responsibilities and patient rights if US Immigration and Customs Enforcement officers (ICE) entered the ED. The regional Immigration Rapid Response Team (a group of attorneys and community groups), ED leadership, and physicians implementing a similar guideline in different clinical settings provided feedback on the educational guideline prior to *Implementation*. The handout was uploaded to E*Drive (accessible at https://edrive.ucsf.edu/immigration-and-customs-enforcement-ice-workflow) and was designed in the same format as other clinical guidelines on E*Drive, which has shown to facilitate ease of access, familiarity, and adherence to standards of care.^12^,^13^

### Immigrant Health Didactic Session

For the *Implementation* of these educational resources and to address Objectives 3–5, we designed a 30-minute didactic session. This was an interactive session (live and unrecorded) with anonymized real-life cases exemplifying the EP’s role in caring for immigrants, including patient-centered documentation practices, hospital ED policies, and the educational resources on E*Drive. An EP-attorney content expert provided feedback on the curriculum prior to *Implementation*. The session was presented annually at the weekly EM residency educational conference, thus incorporating it as an EM competency. For ease of access, the material was also uploaded to E*Drive (accessible at https://edrive.ucsf.edu/immigration-didactics).

## IMPACT

The final step, *Evaluation*, is critical in capturing the curriculum’s impact and informing iterative improvements. In addition to the previously discussed *Evaluation* from community partners, EM leadership, and EM content experts prior to *Implementation*, we developed a Qualtrics survey (Qualtrics International Inc, Provo, UT) for learners with five 5-point Likert scale questions to assess perceived knowledge confidence and three test-style, multiple-choice questions to assess acquired knowledge.^14^,^15^ Questions examined learners’ confidence in their familiarity with immigration-related patient resources (Objective 1); knowledge of the response to ICE in the ED (Objective 2); ability to advocate for and inform immigrant patients of their rights (Objective 3); and understanding of immigration-related hospital policies (Objective 5). The knowledge-based questions assessed understanding of immigration status being HIPPA protected (Objective 4); the risk of documenting immigration status in the chart (Objective 4); and basic information about immigration warrants (Objectives 2 and 5).

The pre-survey was administered to learners before the educational didactic session through email who were reminded at the start of the session. Learners were then asked to complete a post-session survey at the end of the session and reminded again through email. A unique identifier was used to pair the pre- and post-survey responses. Fifty-two EPs completed the pre-survey (20 in 2022, 32 in 2023), and 29 completed the post-survey (18 in 2022, 11 in 2023), with 17 completing both (8 in 2022, 9 in 2023). Ultimately, 47 targeted learners (27%) completed either the pre -or post-survey, and 17 (10%) completed both surveys, for a total response rate of 36%.

**Figure 2:**
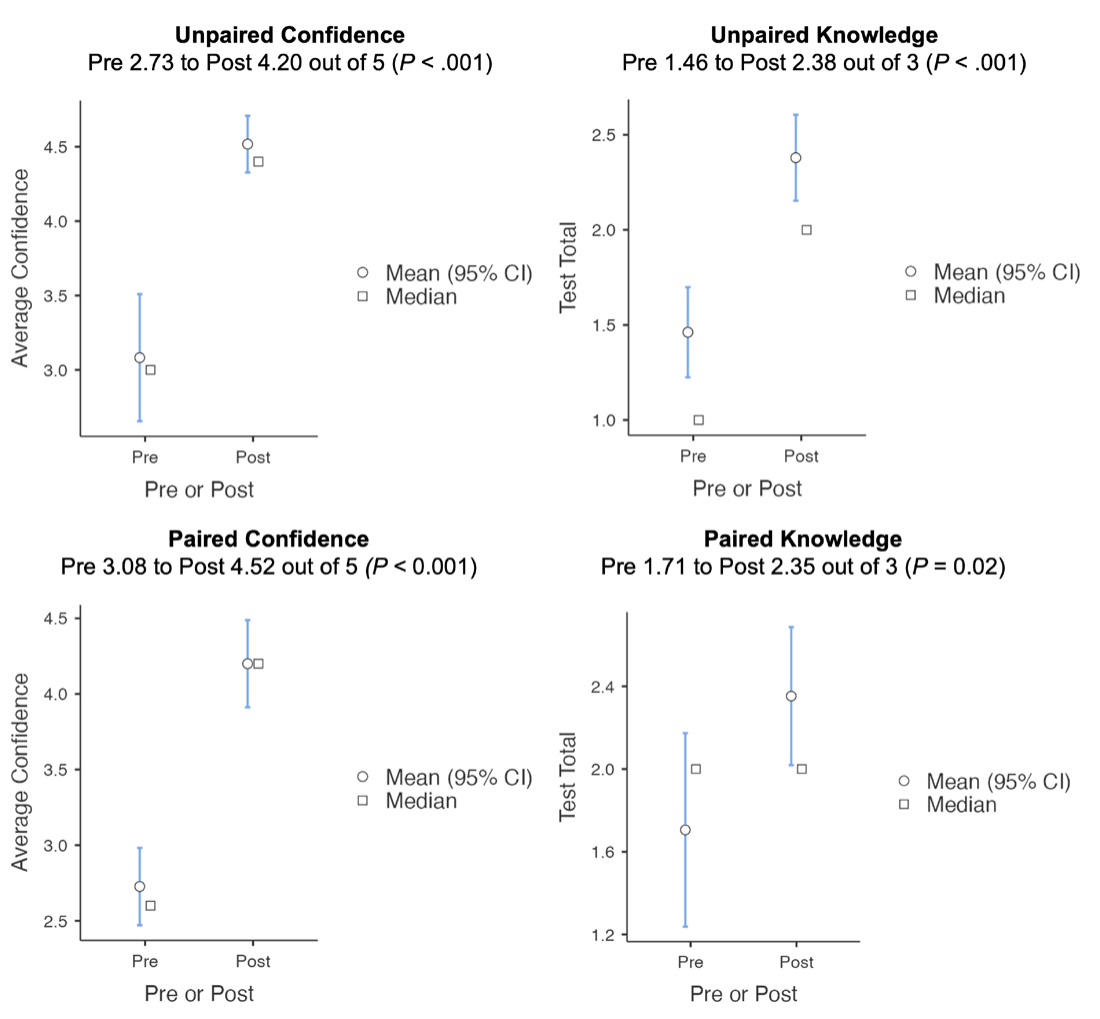
Pre- and post-survey results. *CI*, confidence interval.

We analyzed the pre- and post-survey average confidence and knowledge scores using the Mann-Whitney U test for unpaired responses and the Wilcoxon signed-rank test for paired responses. We additionally compared the pre- and post-survey distributions of training level and individual question responses using the chi-squared test for association (Appendix A). There were no significant differences in training level between the pre- and post-survey responders (*P*=.07). Regarding confidence, there was a significant difference (p<.001) in responses to all of the confidence questions except for the fifth one (p=0.29). In terms of knowledge gained, there was a significant difference in responses to the first two of the three knowledge questions (*P*=.0001, *P*<.001, and *P*=.12, respectively). Overall, we saw a significant increase in overall confidence, with the average score across the five questions on the 5-point Likert scale increasing by 1.47 (*P* < .001) in unpaired responses and by 1.40 (*P* < 0.001) in paired responses, and a significant increase in the percentage of knowledge questions answered correctly by 30.66% (*P* < .001) in unpaired responses and 33% (*P* = .02) in paired responses, meeting the educational objectives (Figure 2).

## LIMITATIONS

There are limitations to this study. First, the educational session did not include all targeted learners, as 100% attendance is not required at the residency educational conference. Second, the paired response sample size was limited, as several learners who completed the pre-survey were unable to ultimately attend. Moreover, although learners were asked to complete both surveys at the didactic session, there were still 12 unpaired post-survey responses. Third, while not a significant difference, there was a greater percentage of faculty members who responded to the pre-survey, potentially accounting for some of the differences. The paired survey responses help to control for this, showing similar improvements in scores. Finally, while our post-session data showed promising results in terms of confidence and knowledge acquisition, it does not adequately assess long-term knowledge retention. Although the small sample size makes it difficult to interpret statistical significance, the data consistently aligns with the Kirkpatrick framework, level 2, showing learners’ confidence and knowledge.^16^

## CONCLUSION

Our study examined the development of immigrant health educational tools for emergency physicians and analyzed their impact. This curriculum design offers a collaborative, interprofessional approach to addressing the knowledge gap among EPs on immigration-related best practices, protocols, and policies. A multi-pronged approach is needed to address various topics within immigrant health (eg, discharge resources to medical-legal policy), accommodate different learning styles (eg, didactics, digital tools, guidelines), and allow for resources that are accessible when needed (eg, on a centralized, open-access clinical information hub such as E*Drive).

Our curriculum can serve as a starting point for EDs looking to improve care for immigrant populations. In designing these interventions, we emphasize the importance of interprofessional community partnership and iterative curriculum design to better ensure relevant and effective interventions. Future studies could assess knowledge retention longitudinally, educational tool utilization and favorability, and assess the impact of these interventions from the patient perspective for long-term conclusions and assessment of higher Kirkpatrick levels.^16^

## Supplementary Information
