# Supplementary material for: Developing Interprofessional Immigrant Health Education for Emergency Physicians
Source: West J Emerg Med. 2025 Jul 12;26(4):781–5. doi: 10.5811/westjem.33576 (PMC12342424; doi:10.5811/westjem.33576)
Supplement: Supplementary file 1 [file wjem-26-781-s001.docx]

| **Unpaired Averages** | | | | | | | |
| --- | --- | --- | --- | --- | --- | --- | --- |
|  | **Mean** | **95% Confidence Interval** | **Median** | **Total Responses (n=)** | **P-Value** | | |
| **Average Confidence (1-5)** |  |  |  |  |  | | |
| Pre | 2.73 | (2.60, 2.86) | 2.60 | 52 | **<0.001** | | |
| Post | 4.20 | (4.05, 4.35) | 4.20 | 29 |  | | |
| **Average Knowledge (0-3)** |  |  |  |  |  | | |
| Pre | 1.46 | (1.34, 1.58) | 1.00 | 52 | **<0.001** | | |
| Post | 2.38 | (2.27, 2.50) | 2.00 | 29 |  | | |
| **Paired Averages** | | | | | | | |
|  | **Mean** | **95% Confidence Interval** | **Median** | **Total Responses (n=)** | **P-Value** | | |
| **Average Confidence (1-5)** |  |  |  |  |  | | |
| Pre | 3.08 | (2.86, 3.30) | 3.00 | 17 | **<0.001** | | |
| Post | 4.52 | (4.42, 4.62) | 4.40 | 17 |  | | |
| **Average Knowledge (0-3)** |  |  |  |  |  | | |
| Pre | 1.71 | (1.47, 1.95) | 2.00 | 17 | **0.015** | | |
| Post | 2.35 | (2.18, 2.52) | 2.00 | 17 |  | | |
| **Demographics** | | | | | | | |
|  | **PGY-1** No. (%) | **PGY-2** No. (%) | **PGY-3** No. (%) | **PGY-4** No. (%) | **Faculty** No. (%) | **P-Value** | |
| **Training Level** |  |  |  |  |  |  | |
| Pre | 8 (15%) | 2 (4%) | 4 (8%) | 7 (13%) | 31 (60%) | 0.069 | |
| Post | 5 (17%) | 1 (3%) | 8 (28%) | 6 (21%) | 9 (31%) |  | |
| **Confidence Questions** | | | | | | | |
|  | **Strongly Agree** No. (%) | **Somewhat Agree** No. (%) | **Neither Agree nor Disagree** No. (%) | **Somewhat Disagree** No. (%) | **Strongly Disagree** No. (%) | **P-Value** | |
| **Q1. I know how to respond if immigration enforcement were to show up in the ED for my patient.** |  |  |  |  |  |  | |
| Pre | 4 (8%) | 10 (19%) | 4 (8%) | 18 (35%) | 16 (31%) | **<0.001** | |
| Post | 10 (34%) | 17 (59%) | 1 (3%) | 0 (0%) | 1 (3%) |  | |
| **Q2. I feel confident in my ability to advocate for my immigrant patients.** |  |  |  |  |  |  | |
| Pre | 5 (10%) | 10 (19%) | 12 (23%) | 18 (35%) | 7 (13%) | **<0.001** | |
| Post | 10 (34%) | 16 (55%) | 1 (3%) | 1 (3%) | 1 (3%) |  | |
| **Q3. I am familiar with the hospital's policies impacting care for immigrant patients.** |  |  |  |  |  |  | |
| Pre | 3 (6%) | 7 (13%) | 3 (6%) | 19 (37%) | 20 (38%) | **<0.001** | |
| Post | 7 (24%) | 19 (66%) | 1 (3%) | 1 (3%) | 1 (3%) |  | |
| **Q4. I am familiar with the hospital's community resources impacting care for immigrant patients.** |  |  |  |  |  |  | |
| Pre | 3 (6%) | 5 (10%) | 7 (13%) | 19 (37%) | 18 (35%) | **<0.001** | |
| Post | 7 (24%) | 19 (66%) | 1 (3%) | 1 (3%) | 1 (3%) |  | |
| **Q5. I believe ED providers have a responsibility to inform immigrant patients of their rights and connect them with local resources.** |  |  |  |  |  |  | |
| Pre | 29 (56%) | 12 (23%) | 7 (13%) | 1 (2%) | 3 (6%) | 0.292 | |
| Post | 21 (72%) | 6 (21%) | 1 (3%) | 0 (0%) | 1 (3%) |  | |
| **Knowledge Questions** | | | | | | | |
| **Q1. Is immigration status protected under HIPAA?** | **Yes** *(Correct)* No. (%) | | **No** No. (%) | | **Not Sure** No. (%) | | **P-Value** |
| Pre | 26 (50%) | | 4 (8%) | | 22 (42%) | | **0.011** |
| Post | 23 (79%) | | 3 (10%) | | 3 (10%) | |  |
| **Q2. Should you document a patient’s immigration status?** | **No, it can put patients at risk and can interfere with patient trust.** *(Correct)* No. (%) | | **Yes, documenting facilitates communication among healthcare team.** No. (%) | | **Maybe, it depends on the situation and provider discretion.** No. (%) | | **P-Value** |
| Pre | 27 (52%) | | 3 (6%) | | 22 (42%) | | **<0.001** |
| Post | 28 (97%) | | 0 (0%) | | 1 (3%) | |  |
| **Q3. What should you do if immigration enforcement is searching for your patient in the ED?** | **Call the AOD (administrator on duty) and ask to see the warrant. They can enter with any search warrant signed by a magistrate judge.** *(Correct)* No. (%) | | **Call the AOD (administrator on duty) and ask to see the warrant. They can enter with any search warrant signed by an immigration judge.** No. (%) | | **After checking their badge, lead them to your patient. They have the right as law enforcement.** No. (%) | | **P-Value** |
| Pre | 23 (44%) | | 29 (56%) | | 0 (0%) | | 0.199 |
| Post | 18 (62%) | | 11 (38%) | | 0 (0%) | |  |
| **Note.** There were 52 pre-survey responses and 29 post-survey responses for unpaired comparisons. There were 17 pre and post-survey responses for paired comparisons. Unpaired averages compared using Mann-Whitney U Test. Paired averages compared using Wilcoxon Signed-Rank Test. Training levels and individual questions compared using Chi-Squared Test. | | | | | | | |
